# A Comparison of Vascular Effects from Complex and Individual Air Pollutants Indicates a Role for Monoxide Gases and Volatile Hydrocarbons

**DOI:** 10.1289/ehp.0901207

**Published:** 2010-03-02

**Authors:** Matthew J. Campen, Amie K. Lund, Melanie L. Doyle-Eisele, Jacob D. McDonald, Travis L. Knuckles, Annette C. Rohr, Eladio M. Knipping, Joe L. Mauderly

**Affiliations:** 1 Toxicology Division, Lovelace Respiratory Research Institute, Albuquerque, New Mexico, USA; 2 Electric Power Research Institute, Palo Alto, California, USA

**Keywords:** atherosclerosis, carbon monoxide, cardiovascular, lipid peroxidation, nitric oxide, particulate matter, vascular remodeling, zymography

## Abstract

**Background:**

Emerging evidence suggests that the systemic vasculature may be a target of inhaled pollutants of vehicular origin. We have identified several murine markers of vascular toxicity that appear sensitive to inhalation exposures to combustion emissions.

**Objective:**

We sought to examine the relative impact of various pollutant atmospheres and specific individual components on these markers of altered vascular transcription and lipid peroxidation.

**Methods:**

Apolipoprotein E knockout (ApoE^−/−^) mice were exposed to whole combustion emissions (gasoline, diesel, coal, hardwood), biogenically derived secondary organic aerosols (SOAs), or prominent combustion-source gases [nitric oxide (NO), NO_2_, carbon monoxide (CO)] for 6 hr/day for 7 days. Aortas were assayed for transcriptional alterations of endothelin-1 (*ET-1*), matrix metalloproteinase-9 (*MMP-9*), tissue inhibitor of metalloproteinase-2 (*TIMP-2*), and heme oxygenase-1 (*HO-1*), along with measures of vascular lipid peroxides (LPOs) and gelatinase activity.

**Results:**

We noted transcriptional alterations with exposures to gasoline and diesel emissions. Interestingly, *ET-1* and *MMP-9* transcriptional effects could be recreated by exposure to CO and NO, but not NO_2_ or SOAs. Gelatinase activity aligned with levels of volatile hydrocarbons and also monoxide gases. Neither gases nor particles induced vascular LPO despite potent effects from whole vehicular emissions.

**Conclusions:**

In this head-to-head comparison of the effects of several pollutants and pollutant mixtures, we found an important contribution to vascular toxicity from readily bioavailable monoxide gases and possibly from volatile hydrocarbons. These data support a role for traffic-related pollutants in driving cardiopulmonary morbidity and mortality.

Emerging evidence from epidemiologic studies suggests that source-specific air pollution may have a focused impact on cardiovascular health. In particular, exposure to traffic has been shown to be a stronger risk for acute myocardial infarction, and proximity to roadways is more strongly associated with coronary artery calcification, than are indices of particulate matter (PM) exposure (Hoffmann et al. 2007; Peters et al. 2004). Such traffic proximity studies suggest that PM of secondary origin, which is typically more regionally distributed, does not drive these cardiovascular sequelae. Although PM has a definite toxic effect on the systemic vasculature in rodent models and in controlled human studies (Shah et al. 2008; Sun et al. 2005), environmental exposure to PM never occurs without concomitant exposure to numerous gaseous copollutants. Thus, the strong statistical signal observed from traffic-related exposures may reflect a cumulative impact of fresh vehicular emissions rather than toxicity of individual pollutants.

Diseases of the systemic vasculature can manifest in many ways, and we have a growing appreciation that PM air pollution may exacerbate atherosclerosis (Araujo et al. 2008), hypertension (Sun et al. 2008), and diabetic vasculopathy (Sun et al. 2009). Although the spectrum of vascular disorders is wide, numerous common elements drive progression of disease, such as vascular inflammation, enzymatic remodeling of the extracellular matrix, and lipid oxidation and deposition. We have shown that whole gasoline engine combustion emissions can promote activity of metalloproteinases (MMPs), increase vascular lipid peroxidation, and initiate mRNA transcription of MMPs, endothelin-1 (*ET-1*), and heme oxygenase (*HO-1*) (Lund et al. 2007, 2009). More recently, we observed that diesel emissions exposure can induce similar findings along with vascular inflammation and collagen deposition (Campen et al. 2010). Combined, such responses provide a platform for a more detailed inquiry into the roles of specific pollutants in driving the responses to complex mixtures.

Although our previous studies have shown a putative role of gasoline exhaust in cardiovascular toxicity, we have not previously extended the analysis to other important components of ambient air place the gasoline exhaust results in context. To address this, the present study performed head-to-head comparisons of the effects of several important environmental mixtures and complementary studies of putative gases. More than simply looking at the magnitude of toxicity, we were interested in the degree to which similar atmospheres and individual components thereof could recapitulate biological effects. Exposure atmospheres included gasoline and diesel engine exhaust, hardwood smoke, a simulated “downwind” coal combustion atmosphere (SDCCA), biogenically derived secondary organic aerosols (SOAs), and individual combustion source gases [nitric oxide (NO), nitrogen dioxide (NO_2_), carbon monoxide (CO)]. We used a well-characterized model of vascular toxicity, the apolipoprotein E knockout (ApoE^−/−^) mouse, to assess comparative responses to these atmospheres. The findings from this study indicate a complex response pattern that is consistent with certain components of the complex emissions, but we could not completely recreate the gestalt vascular impact of vehicular exhaust by using individual components.

## Materials and Methods

### Animals

Adult, male ApoE^−/−^ mice (8 weeks old), on a C57BL/6J background, were obtained from a commercial vendor (Taconic, Oxnard, CA). Upon arrival, mice were fed a high-fat diet (Harlan Teklad #88137; Harlan Teklad, Madison, WI) *ad libitum*, beginning 30 days before inhalation exposure. Mice were housed in an Association for Assessment and Accreditation of Laboratory Animal Care–approved facility throughout the study, except during exposures to SOAs. For other exposures, the mice were housed in shoebox cages placed in whole-body inhalation exposure chambers (H2000; Hazleton Systems, Maywood, NJ) with the cage filter covers and chow removed for the daily exposure periods.

Subject numbers were generally between 8 and 10 for each exposure/assay but may have varied from as few as 5 to as many as 14 for certain assays. Because of either inadequate sample collection or RNA quality, occasional samples were discarded from analysis. For complete clarity, Supplemental Material, Table 1 (10.1289/ehp.0901207) provides explicit information on subject numbers and results (mean ± SD) for all exposures and assays. Animals used in this study were treated humanely and with regard to alleviation of suffering. All animal procedures were conducted with full approval of the Lovelace Respiratory Research Institute’s Animal Care and Use Committee.

### Exposures

The mice were exposed 6 hr/day for 7 consecutive days, using several exposure systems that were previously characterized in detail for studies by the National Environmental Respiratory Center (http://www.nercenter.org) and related programs. The diesel exhaust, SDCCA, hardwood smoke and SOA exposures were conducted at matching PM concentrations (300 μg/m^3^ for the present study). Because gasoline exhaust contains a very low mass of PM, mice were exposed to the highest concentration used previously (60 μg PM/m^3^) (Lund et al. 2007, 2009).

Whole, diluted diesel and gasoline exhausts were generated from engines on test stands and operated on variable-load duty cycles. Diesel exhaust was generated from 2000 model 5.9-L Cummins engines burning 300 ppm sulfur certification fuel and operated on the federal heavy-duty test cycle (McDonald et al. 2004). Gasoline exhaust was generated from 1996 model General Motors 4.3-L engines burning fuel blended to 2001–2002 national average specifications for regular unleaded, nonoxygenated fuel and operated on the California Unified Driving Cycle (McDonald et al. 2008). Hardwood smoke was generated by burning split oak in an uncertified stove operated on a daily three-phase home heating cycle (McDonald et al. 2006). A mixture (SDCCA) simulating key components of emissions from a coal-fired power plant at downwind locations was generated by burning western low-sulfur sub-bituminous coal in an electric furnace and adding sulfate and gases to achieve the desired mixture (Seagrave et al. 2008).

The two SOA atmospheres were generated in a continuous flow stir reaction chamber as described by McDonald et al. (2010), with the exception that the ozone and components of nitrogen oxides (NO_x_) of the gaseous fraction of the atmosphere were removed using a spent honeycomb carbon denuder. The denuder removed ozone and most NO_x_ while allowing the hydrocarbon portion to pass through. The two SOA atmospheres are referred to here as the neutral (NO + α-pinene) or acidic [NO + α-pinene + sulfur dioxide (SO_2_)] atmospheres. Although both atmospheres had some acidity due to the presence of organic acids, the acidic atmosphere was distinguished by the presence of the SO_2_ that oxidized to sulfuric acid and led to formation of organosulfate compounds. Mice were exposed whole body in metal cages to a continuous stream drawn from the reaction chamber. After daily exposures, mice were returned to home cages.

The gas atmospheres were generated by diluting NO, CO, and NO_2_ from commercially available tanks with room air. Concentrations were monitored by chemiluminescence. Table 1 presents the concentrations of PM, NO_x_ and CO, as well as total nonmethane hydrocarbons, in all atmospheres. Levels of gaseous pollutants were chosen to match the highest levels in gasoline emissions, which were higher than all other exposure atmospheres.

Individual control groups were included for each combustion and SOA atmosphere. Gas exposures were conducted in parallel over two periods, so two control groups of 10 mice each were pooled for these comparisons.

### Plasma and tissue collection

Approximately 18 hr after cessation of the seventh exposure, ApoE^−/−^ mice were anesthetized with Euthasol (390 mg pentobarbital sodium, 50 mg phenytoin sodium/mL; VIRBAC, Ft. Worth, TX) diluted 1:10 and administered at a dose 0.1 mL per 30 g mouse, and euthanized by exsanguination. Blood was collected in a heparinized syringe (Becton Dickinson Vacutainer Systems, Franklin Lakes, NJ) by cardiac puncture and immediately centrifuged (950 × *g*, 10 min, 4°C) to separate plasma. Additionally, the aorta and heart were dissected, weighed, and frozen in liquid nitrogen. Tissue was stored at − 80°C until assayed.

### Real-time polymerase chain reaction

Total RNA was isolated from the aortic arch as previously described (Lund et al. 2007), using RNeasy Fibrous Tissue Mini Kit (Qiagen, Valencia, CA). Real-time polymerase chain reaction (PCR) was performed using an iCycler (Biorad, Hercules, CA) and an ABI 7500 (Applied Biosystems, Foster City, CA).

### Aortic lipid peroxides (TBARS assay)

To assess vascular oxidative stress, lipid peroxidation was assessed using a thiobarbituric acid reactive substances (TBARS) assay, as previously described (Lund et al. 2007). The descending aorta was resuspended by diluting 1:10 (wt/vol) in normal saline and then homogenized and sonicated for 15 sec at 40 kHz; homogenates were used to determine TBARS levels as described below. A TBARS assay kit (OXItek; ZeptoMetrix Corp., Buffalo, NY) was used to measure lipid peroxide (LPO) levels in whole tissue homogenates. Duplicate samples were read on a spectrophotometer (Lambda 35; Perkin Elmer, Boston, MA) and using a malondialdehyde (MDA) standard curve, and results expressed as MDA equivalents.

### *In situ* zymography

Zymography to assess activity of MMP-2/9 in aortas was conducted as previously described (Lund et al. 2009). Briefly, aorta cryosections were incubated with a dye quenched-gelatin (EnzChek; Molecular Probes, Invitrogen, Carlsbad, CA) and 1 μg/mL 4′,6-diamidino-2-phenylindole (DAPI; nuclei stain, Invitrogen) for 6 hr in a dark, humid chamber at 37°C. After incubation, green fluorescent staining for all regions of the intima and media was quantified using Image J software (version 1.4; National Institutes of Health, Bethesda, MD).

### Statistical analysis

Statistical comparisons were restricted to concomitantly exposed groups, which reduces variations from litter, transportation, and other factors outside of our control. For combustion emissions exposures (e.g., filtered air vs SDCCA) and SOA exposures, a Student *t*-test was used to compare PCR, LPO, and gelatinase activity data. For statistical comparisons among multiple (i.e., dose response with gas exposures) groups, a one-way analysis of variance (ANOVA) was used (GraphPad Prism, version 5.01; GraphPad, La Jolla, CA). To identify not only group differences but also dose–response relationships, two post hoc tests were applied in these conditions: a Bonferroni multiple comparison test and a linear trend test. A value of *p* < 0.05 was considered statistically significant. Results were examined for sensitivity to assumptions of normality, and few deviations were found. In those few cases, the results were not sensitive to the discrepancies with the underlying assumptions.

## Results

### Complex emissions

**Figure 1:**
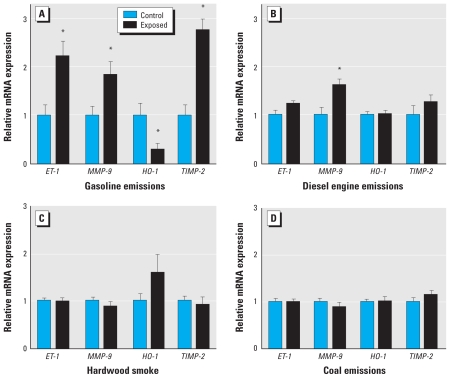
Changes in aortic mRNA transcript abundance for *ET-1*, *MMP-9*, *HO-1*, and *TIMP-2* from ApoE^−/−^ mice after 7-day exposure to gasoline engine emissions (*A*), diesel engine emissions (*B*), hardwood smoke (*C*), or SDCCA (*D*). Data are mean ± SE. *Significant difference from control by two-tailed Student’s *t*-test (*p* < 0.05).

Consistent with previous studies of both 7- and 50-day exposures (Lund et al. 2007, 2009), gasoline emissions caused a significant up-regulation of several aortic mRNA biomarkers of vascular remodeling, including *MMP-9*, *ET-1*, and tissue inhibitor of metalloproteinase (*TIMP-2*) (Figure 1A). The level of increase was two to three times control levels. Interestingly, aortic *HO-1* mRNA was significantly down-regulated (*p* = 0.036) by gasoline exposure, compared with filtered air controls, which was qualitatively different from subchronic exposures (Lund et al. 2007), suggesting an as yet unexplained pathophysiologic adaptation. Diesel emissions also induced a significant up-regulation of *MMP-9*, at a level similar to that induced by gasoline emissions (Figure 1B). Although we noted a slight increasing trend in *ET-1*, this effect was not significant (*p* = 0.068). However, neither the hardwood smoke (Figure 1C) nor SDCCA (Figure 1D) caused significant alterations in the four PCR products that we interrogated, although we noted an increasing trend for *HO-1* in hardwood smoke–exposed mice (*p* = 0.1).

**Figure 2:**
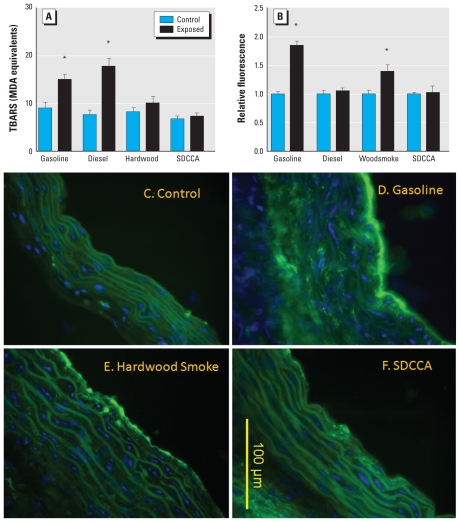
Lipid peroxidation (*A*) and MMP activity (*B*) in aortas from ApoE^−/−^ mice exposed to gasoline engine emissions, diesel engine emissions, hardwood smoke, or SDCCA. Data are mean ± SE. (*C*–*F*) Representative images of aortic gelatinase (MMP-2/9) activity, as assessed by *in situ* zymography. Green autofluorescence is apparent in lamellar structures along with blue nuclear contrast staining (DAPI). Compared with control (*C*), increased intimal and medial green fluorescence is apparent in aortic sections from ApoE^−/−^ mice exposed to gasoline (*D*) and hardwood smoke (*E*) but not to SDCCA (*F*). *Significant difference from control by two-tailed Student’s *t*-test (*p* < 0.05).

Aortic lipid peroxidation was significantly enhanced by both gasoline and diesel engine emissions (Figure 2A). The effect from the diesel emissions was slightly greater in magnitude than that from gasoline engine emissions, although this difference was not statistically significant. Hardwood smoke and SDCCA atmospheres caused no significant change in aortic LPOs.

To ascertain induction of aortic gelatinase activity, we incubated cryosections of aorta with a dye-quenched gelatin, as previously described (Lund et al. 2009). Consistent with previous studies (Lund et al. 2009), compared with controls (Figure 2B,C), gasoline emissions caused a significant increase (~ 80%) in gelatinase activity in the aortas of ApoE^−/−^ mice (Figure 2B,D). Hardwood smoke, interestingly, induced a roughly 40% increase in gelatinase activity-related fluorescence compared with control aortas (Figure 2B,E). Diesel emissions and SDCCA caused no significant change in gelatinase activity (Figure 2B,F).

### Secondary organic aerosols

**Figure 3:**
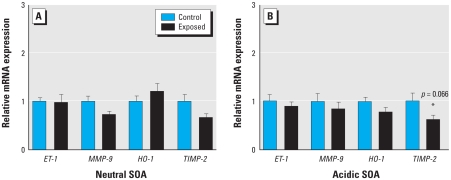
Changes in aortic mRNA transcript abundance for *ET-1*, *MMP-9*, *HO-1*, and *TIMP-2* after 7-day exposure to the neutral (*A*) and acidic (*B*) SOA atmospheres. Data are mean ± SE.

**Figure 4:**
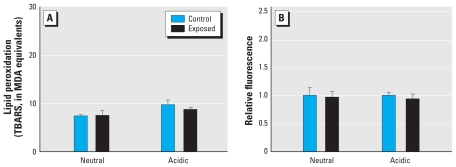
Lipid peroxidation (*A*) and MMP-2/9 activity (assessed by *in situ* zymography; *B*) in aortas from ApoE^−/−^ mice after 7-day exposure to the acidic and neutral SOA atmospheres. Data are mean ± SE.

We generated two SOAs, at concentrations of 300 μg PM/m^3^. Residual gases were contained in the atmospheres, but at quite low levels (NO = 200 ppb). Similar to previous studies at lower concentrations (McDonald et al. 2010), neither the neutral nor the acidic SOA induced increases in any parameter related to vascular toxicity, although we noted a nonsignificant decreasing trend for *TIMP-2* mRNA for both particle models (Figure 3A,B). We observed no effects on aortic lipid peroxidation (Figure 4A) or gelatinase activity (Figure 4B).

### Principal gases (NO, CO, NO_2_)

**Figure 5:**
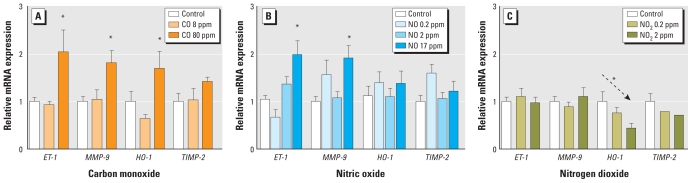
Changes in aortic mRNA transcript abundance for *ET-1*, *MMP-9*, *HO-1*, and *TIMP-2* from ApoE^−/−^ mice after 7-day exposure to CO (*A*), NO (*B*), and NO_2_ (*C*), across several concentration levels. Data are mean ± SE. *Significant difference from control by one-way ANOVA. Arrow (*C*), statistically significant post hoc test for linear trend (*p* < 0.05).

Because the most prominent gases by mass concentration in gasoline and diesel engine emissions were CO, NO, and NO_2_, we specifically studied these gases to determine if any could replicate the vascular responses observed from exposure to whole exhausts. Indeed, CO exposure for 7 days at 80 ppm led to significant elevations in *ET-1* and *MMP-9*, but there appeared to be no effect or even trend at the 8-ppm level (Figure 5A). The net magnitude of effect, a 100% increase, was remarkably similar to the effect induced by gasoline emissions. NO, another freely diffusible monoxide gas, also induced a doubling of *ET-1* and *MMP-9* mRNA (Figure 5B). Neither CO nor NO altered *HO-1* or *TIMP-2*. Interestingly, NO_2_ caused a dose-dependent reduction in aortic *HO-1* (significant only by linear trend test; Figure 5C) but did not affect any other PCR product.

**Figure 6:**
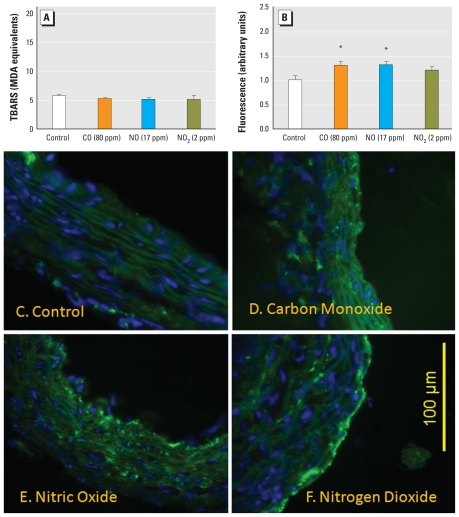
Lipid peroxidation (*A*) and MMP-2/9 activity (assessed by *in situ* zymography; *B*) in aortas from ApoE^−/−^ mice after 7-day exposure to CO (80 ppm), NO (17 ppm), or NO_2_ (2 ppm). Data are mean ± SE. (*C*–*F*) Representative images of aortic gelatinase (MMP-2/9) activity, as assessed by *in situ* zymography. Green autofluorescence is apparent in lamellar structures along with blue nuclear contrast staining (DAPI). Compared with control (*C*), increased intimal and medial green fluorescence is apparent in aortic sections from ApoE^−/−^ mice exposed to CO (*D*) and NO (*E*) but not NO_2_ (*F*). *Significant difference from control by one-way ANOVA, with a post hoc test for linear trends (*p* < 0.05).

We observed no significant change in aortic LPO for any of the principal gases (Figure 6A). The control values in these assays were consistently lower than those in the other exposure situations; however, this is most likely related to differences in the standard curves, generated separately for each individual assay. Monoxide exposure caused a significant increase in the relative activity of vascular gelatinases (Figure 6B–F). We did not use an absolute quantitative approach, but by comparing the relative fluorescence in cross sections of aortas, we noted that, compared with controls (Figure 6C), CO and NO each induced an approximately 30% increase of activity (Figure 6B,D,E). NO_2_ exposure was associated with a middling response that was not statistically significant (Figure 6B,F). Despite the qualitative nature of these assays, negative findings for SDCCA, diesel emissions, and SOAs provide confidence that such responses are meaningful and likely explain a portion of the effects of the gasoline atmospheres.

### Results summary

Table 2 summarizes all effects across the various pollutant atmospheres; Supplemental Table 1 (doi:10.1289/ehp.0901207) provides detailed findings. *HO-1* was generally unresponsive to all atmospheres, despite previous observations that extended (50-day) gasoline emissions exposures caused a significant up-regulation (Lund et al. 2007). Indices of vascular remodeling (*MMP-9*, *ET-1*, and *TIMP-2*) were most noticeably altered by the gasoline emissions atmosphere and, to a lesser extent, diesel. These effects were recapitulated by exposure to CO at 80 ppm, equivalent to the level in gasoline emissions. NO induced some changes in vascular remodeling indices but not as potently as did CO. Lipid peroxidation, interestingly, was induced only by the vehicular emissions, with diesel appearing slightly more potent than gasoline emissions. This effect was not reproduced by either PM (SOAs) or principal gases (NO_x_, CO), suggesting a role for either hydrocarbons or a more complex gas–particle relationship. Gelatinase activity was induced by gasoline emissions and, surprisingly, hardwood smoke, along with the monoxide gases CO and NO.

## Discussion

In the present study, vehicular emissions (gasoline and diesel) induced a complex panel of systemic vascular responses, whereas other complex environmental mixtures (hardwood smoke, SDCCA, neutral and acidic biogenic SOAs) elicited little or no response in the assays examined. The potency of vehicular emissions is consistent with numerous epidemiologic reports of traffic-related exacerbations of cardiovascular disease (Peters et al. 2004; Sarnat et al. 2008). Moreover, although certain aspects of the vascular response could be recreated by monoxide gases, the aortic lipid peroxidation induced by vehicular emissions was not reproducible by such exposures. Additionally, activation of MMPs could be driven by monoxides, gasoline emissions, and hardwood smoke; because hardwood smoke was virtually devoid of NO_x_ and relatively low in CO, this suggests an effect common to various combustion species. The results of these head-to-head comparisons indicate, not surprisingly, that complex air pollution mixtures drive complex pathophysiologic responses.

In general, we have focused on a profile of parameters related to atherosclerosis progression. Because of the short-term nature of the exposures (7 days), we did not assess plaque progression histopathologically, instead opting for these more sensitive and global markers. In a recent 50-day diesel exposure study, we found reasonable correlation among plaque inflammation and collagen deposition and *MMP-9*, *ET-1*, and TBARS. However, the net growth of the atheromatous lesion was not significantly affected by this subchronic exposure (Campen et al. 2010). The acute responses of *ET-1* and *MMP-9* are also somewhat conserved in humans after controlled exposures to diesel emissions (Lund et al. 2009). MMP-9 and gelatinase activity have been shown to cause a destabilization of advanced plaques in mouse models, and in humans MMP-9 is a biomarker for risk of acute coronary events (Gough et al. 2006; Hlatky et al. 2007). Similarly, lipid peroxidation is a known driver of the inflammatory pathways that are central to atherosclerosis (Chou et al. 2009). We also examined *HO-1*, ostensibly as a marker of oxidative stress; however, we found this marker to be poorly responsive and did not correlate with TBARS or any other end point. *TIMP-2* was studied primarily as a marker that was highly up-regulated in earlier studies with gasoline emissions (Lund et al. 2007) and is known to have a broad involvement in cardiovascular disease (Johnson et al. 2006). Thus, in the present study, atmospheres that increase these biomarkers would, in theory, be able to promote atherosclerotic pathologies or worsen outcomes.

Our findings indicate a substantial biological role for the gaseous components of whole emissions. CO exposure alone, at 80 ppm, was capable of recreating the transcriptional effects of whole vehicular emissions, causing significant increases in aortic *MMP-9*, *ET-1*, and *HO-1*. With no clear effect at 8 ppm, we have some basis for a no observed effects level. This level closely matches current regulated ambient levels, although the toxicodynamics of this phenomenon requires a study with greater precision and better characterization of concentration response and the temporal nature of effects in order to extrapolate with confidence to potential environmental effects. Additionally, we cannot conclude that the ApoE^−/−^ mouse is uniquely susceptible to CO, and comparative exposures with other strains/species is essential for proper context.

In our previous studies with gasoline emissions, we removed PM by filtration and were unable to reduce the vascular responses (Lund et al. 2007). More recently, a similar study with diesel emissions that consisted of much higher PM levels found a partial role for PM in driving some of the histopathologic responses (Campen et al. 2010). The present findings suggest that a significant portion of the overall vascular induction of aortic *MMP-9* and *ET-1* mRNA from vehicular emissions can be driven by the two readily absorbed monoxide gases, NO and CO. Gasoline emissions exposure led to an approximate doubling of *ET-1* and *MMP-9*, and both CO and NO induced a similar magnitude effect for both mRNA markers. Interestingly, *HO-1* was previously found to be responsive to subchronic gasoline exhaust exposure (Lund et al. 2007), whereas in the present 7-day exposure the response was reduced. Much of the mechanism of action for these changes remains uncertain. Recent findings with whole diesel emissions suggest that endothelial cells may be a specific target for inducing vascular dysfunction, possibly through the inactivation of endothelial NO synthase (Cherng et al. 2009; Knuckles et al. 2008). It is intriguing that NO and CO are both endogenously generated, by NO synthase and HO-1, respectively, and may share certain roles in physiologic homeostasis; there is currently much speculation regarding the interaction between these pathways (Chung et al. 2008).

It should be kept in mind that typical NO measurements at U.S. Environmental Protection Agency monitoring sites rarely exceed 0.2 ppm. However, Fujita et al. (2003) have observed averaged NO levels approaching 0.4 ppm in specific traffic situations. Occupational levels rarely exceed approximately 0.5 ppm in hospitals where inhaled NO may be used therapeutically (Markhorst et al. 1996; Qureshi et al. 2003). Prevalent in fresh vehicular exhaust, NO is highly reactive and almost entirely transformed to other chemical species at the distance of most monitoring stations from major roadways. Thus, in our gasoline engine work, the NO:NO_2_ ratio is much higher than ambient. On one hand, this may reflect some level of artifice in our laboratory-based system; on the other hand, this finding may offer an important clue regarding the relationship between roadway/traffic exposures and adverse cardiovascular sequelae. Ambient NO_x_ measurements are likely to be a poor reflection of roadway or in-cabin vehicle concentrations owing to the rapid transformation of emissions with distance from the roadway. Restrepo et al. (2004) found that although concentrations of PM and a few other criteria pollutants were fairly consistent between their experimental roadway monitors and municipal monitoring stations, levels of CO and NO_2_ were roughly 75% and 140% greater near roadside, respectively. A study of tunnel pollution noted extremely high levels of NO, with mean values roughly 1.3 ppm (De Fré et al. 1994). Similar reports of “hot spots” for CO in tunnels (Kamei and Yanagisawa 1997) and tollbooths (Niza and Jamal 2007) suggest that vehicular sources contribute higher levels of monoxides than is commonly appreciated, although such extremes are likely to be quite rare under current regulatory standards in the United States.

Monoxide gases could recapitulate only a portion of the zymographic activity observed with gasoline emissions and wood smoke. When examining the differences among the atmospheres, volatile hydrocarbons stand out as a component that is especially high in gasoline, less in wood smoke, and virtually absent in SDCCA and diesel. The constituents in this class of chemicals numbers in the hundreds (McDonald et al. 2008), thus identifying the putative culprit(s) would be extremely difficult, experimentally, and at best we can only speculate on the characteristics of drivers. Moreover, although we have conclusively determined that monoxide gases cause this effect, a representative exposure to the milieu of hydrocarbons may not be feasible. Wood smoke has been shown to induce pulmonary MMP activity (Ramos et al. 2009), and a highly reactive component of combustion atmospheres, acrolein, was shown to activate aortic MMPs when administered in chow (O’Toole et al. 2009). As mentioned, the importance of aortic gelatinase activity relates to a potential role in destabilizing vulnerable plaques (Gough et al. 2006), which may be a predisposing factor for acute myocardial infarction. Previous studies with gasoline emissions found a significant effect as early as a single day after exposure (Lund et al. 2009). The details of the relationship between hydrocarbons and vascular MMP activity, and the clinical significance thereof, remain to be elucidated.

Although numerous toxicologic studies of various air pollutants have been reported, rarely are head-to-head comparisons available for *in vivo* assessments. Several reports have compared the toxicities of different PM samples and, using associative statistical analysis, identified putative drivers of the toxic responses. Seagrave et al. (2006) found that the pulmonary toxicity from numerous ambient PM_2.5_ samples, when intratracheally instilled, was most severe in samples with a large contribution of diesel- and gasoline-engine–derived PM. Gerlofs-Nijland et al. (2007) found that pulmonary toxicity was strongest from PM collected near high-traffic areas. Trends for zinc, barium, potassium, and copper were noted, although the authors acknowledge that these may be markers rather than drivers of toxicity. In a recent study, Seagrave et al. (2008) reported on comparative toxicity of the gasoline engine exhaust atmosphere reported here, along with road dust and SDCCA created with two different coal types. That study assessed pulmonary and systemic oxidant potential, lung lavage cell infiltration, and respiratory parameters in healthy rats. Results showed oxidant potential in gasoline exhaust only, with some mild and transient effects in macrophage infiltration and respiration rate in road dust–exposed and SDCCA-exposed animals. McDonald et al. (2008) reported previously on the neutral and acidic SOAs at a slightly lower concentration (200 μg PM/m^3^) and with nearly complete removal (“scrubbing”) of the gas phase. That study showed mild responses in *HO-1* and *MMP-9* in the aorta of ApoE^−/−^ mice, which were not apparent in the present study when the gas phase was included. To our knowledge, our study is the first to address this question from a whole-exhaust perspective, and necessarily with whole-body inhalation exposures. Perhaps consistent with the instillation studies, we note a trend in systemic toxicity from diesel and gasoline engine exposures, although our previous studies (Lund et al. 2007) and follow-up work with gaseous monoxides suggest that PM has a minor effect on the pathways studied.

Although the PM generated in the present study had very different physicochemical profiles, the absence of effects from the SDCCA and SOA atmospheres, combined with previous results from the PM-filtered gasoline exhaust atmosphere (Lund et al. 2007), suggests that the biological pathways in question are not especially sensitive to PM. Of course, only a limited number of PM subtypes have been examined with this animal model. However, despite the robust nature of the observed vascular responses to vehicular emissions and principal gases, it must be kept in mind that the assays we are investigating are limited in pathophysiologic scope. That is to say, we have not assessed vasoactivity changes, or impacts on diabetic/metabolic complications, or numerous other disease pathways that are clearly altered by PM exposure (Araujo et al. 2008; Sun et al. 2005, 2009). Our results, therefore, should not be construed as refutation of numerous other reports, but rather as additional insights into the complexities of the biological impact of combustion-source mixtures.

Along with the limited scope of assays, the translational value of the animal model must be considered as a potential limitation of the present study. The ApoE^−/−^ mouse, on a high-fat diet, manifests cholesterol levels in excess of 1,200 mg/dL and develops vascular fatty streaks and atheromatous plaques extremely rapidly (Zhang et al. 1992). It is unclear whether the air pollution responses we have observed in the aortas of these mice are conserved in humans. Also unclear is the extent to which preexisting vascular disease contributes to the severity of this response. In a previous study in healthy human subjects and ApoE^−/−^ mice, we found a number of parallel biomarkers that were up-regulated after engine emissions exposure, including *ET-1* and *MMP-9* (Lund et al. 2009). We contend, therefore, that at least a portion of this response is conserved and highly relevant to human health and may provide clues as to the relationship between air pollution exposure and the progression of atherosclerosis pathways. Further linkages between this mouse model of disease, healthy mice, healthy humans, and diseased humans simply do not exist at present but are clearly justifiable for future research. Controlled human exposures to these sorts of pollutant atmospheres, with the current knowledge of biological markers of vascular stress, would provide invaluable information for risk assessment. As is true for all laboratory research, it should be noted that these findings and conclusions are limited to the exposure conditions, health end points, and animal model that were used in this study. Also, the vehicle exhausts were generated from decade-old engine technologies and fuels, wood smoke was generated from an uncertified stove, and the exposure levels encompassed by this report were higher than widespread environmental concentrations. Last, although the data presented are based on studies with complex atmospheres, the study design and analysis of results are relatively facile when considering the number of chemical components in the combustion mixtures. Future research should endeavor to incorporate multivariate analyses with more complex study designs to better delineate the putative drivers of vascular toxicity.

In summary, we examined a profile of vasculotoxic responses to combustion-source emissions, principal combustion gases, and SOAs and found that monoxide gases and potentially volatile hydrocarbon species appear to be likely drivers of specific outcomes. However, vascular lipid peroxidation, which is a marker of oxidative stress and a potential promoter of vascular disease, was elevated only by the whole vehicular emissions. These findings offer a compelling parallel with recent epidemiologic reports that indicate an adverse impact of traffic-related exposures.

## Figures and Tables

**Table 1:** Mean concentrations of particle mass, CO, NO_x_, and hydrocarbons in exposure atmospheres.

Pollutant	Particle mass (μg/m^3^)	CO (ppm)	NO (ppm)	NO_2_ (ppm)	Total hydrocarbons (nonmethane; mg/m^3^)
Gasoline exhaust	59.1	103.9	16.7	1.1	15.9
Diesel engine exhaust	319.9	10.2	18	0.9	0.6
Hardwood smoke	319.7	4.0	0	0	1.4
SDCCA	313.4	0.04	0.18	0.1	< 0.1
SOA, acid	280.0	NA	< 0.01	< 0.01	NA
SOA, neutral	306.1	NA	< 0.01	< 0.01	NA
CO	NA	8/80	NA	NA	NA
NO	NA	NA	0.2/2/17	NA	NA
NO_2_	NA	NA	NA	0.2/2	NA

NA, data not available, but presumed no higher than filtered air controls. For CO and NO_x_, targets for different concentrations are shown separated by a slash (/); all CO/NO/NO_2_ concentrations were within 1% of target.

**Table 2:** Qualitative summary of effects across pollutant atmospheres in the present study.

Pollutant	Remodeling markers	Lipid peroxidation	Gelatinase activity
Gasoline exhaust	+++	++	++++
Diesel engine exhaust	+	++	−
Hardwood smoke	−	−	++
SDCCA	−	−	−
Biogenic SOA	−	−	−
CO	+++	−	+
NO	+	−	+
NO_2_	−	−	−

Remodeling markers refer to PCR end points for *MMP-9*, *TIMP-2*, and *ET-1*; lipid peroxidation refers to TBARS assays; gelatinase activity refers to results for *in situ* zymography. Minus signs (−) indicate no meaningful change; plus signs (+) indicate significant effects, increasing in potency with the number of plus signs.

## References

[b1-ehp-118-921] Araujo JA, Barajas B, Kleinman M, Wang X, Bennett BJ, Gong KW (2008). Ambient particulate pollutants in the ultrafine range promote early atherosclerosis and systemic oxidative stress. Circ Res.

[b2-ehp-118-921] Campen MJ, Lund AK, Knuckles TL, Conklin DJ, Bishop B, Young D (2010). Inhaled diesel emissions alter atherosclerotic plaque composition in ApoE^−/−^ mice. Toxicol Appl Pharmacol.

[b3-ehp-118-921] Cherng TW, Campen MJ, Knuckles TL, Gonzalez-Bosc L, Kanagy NL (2009). Impairment of coronary endothelial cell ET_B_ receptor function following short-term inhalation exposure to whole diesel emissions. Am J Physiol Regul Integr Comp Physiol.

[b4-ehp-118-921] Chou MY, Fogelstrand L, Hartvigsen K, Hansen LF, Woelkers D, Shaw PX (2009). Oxidation-specific epitopes are dominant targets of innate natural antibodies in mice and humans. J Clin Invest.

[b5-ehp-118-921] Chung HT, Choi BM, Kwon YG, Kim YM (2008). Interactive relations between nitric oxide (NO) and carbon monoxide (CO): heme oxygenase-1/CO pathway is a key modulator in NO-mediated antiapoptosis and anti-inflammation. Methods Enzymol.

[b6-ehp-118-921] De Fré R, Bruynseraede P, Kretzschmar JG (1994). Air pollution measurements in traffic tunnels. Environ Health Perspect.

[b7-ehp-118-921] Fujita EM, Campbell DE, Zielinska B, Sagebiel JC, Bowen JL, Goliff WS (2003). Diurnal and weekday variations in the source contributions of ozone precursors in California’s South Coast Air Basin. J Air Waste Manag Assoc.

[b8-ehp-118-921] Gerlofs-Nijland ME, Dormans JA, Bloemen HJ, Leseman DL, John A, Boere F (2007). Toxicity of coarse and fine particulate matter from sites with contrasting traffic profiles. Inhal Toxicol.

[b9-ehp-118-921] Gough PJ, Gomez IG, Wille PT, Raines EW (2006). Macrophage expression of active MMP-9 induces acute plaque disruption in apoE-deficient mice. J Clin Invest.

[b10-ehp-118-921] Hlatky MA, Ashley E, Quertermous T, Boothroyd DB, Ridker P, Southwick A (2007). Matrix metalloproteinase circulating levels, genetic polymorphisms, and susceptibility to acute myocardial infarction among patients with coronary artery disease. Atherosclerotic Disease, Vascular Function and Genetic Epidemiology (ADVANCE) Study. Am Heart J.

[b11-ehp-118-921] Hoffmann B, Moebus S, Möhlenkamp S, Stang A, Lehmann N, Dragano N (2007). Residential exposure to traffic is associated with coronary atherosclerosis. Circulation.

[b12-ehp-118-921] Johnson JL, Baker AH, Oka K, Chan L, Newby AC, Jackson CL (2006). Suppression of atherosclerotic plaque progression and instability by tissue inhibitor of metalloproteinase-2: involvement of macrophage migration and apoptosis. Circulation.

[b13-ehp-118-921] Kamei M, Yanagisawa Y (1997). Estimation of CO exposure of road construction workers in tunnel. Ind Health.

[b14-ehp-118-921] Knuckles TL, Lund AK, Lucas SN, Campen MJ (2008). Diesel exhaust exposure enhances venoconstriction through uncoupling of eNOS. Toxicol Appl Pharmacol.

[b15-ehp-118-921] Lund AK, Knuckles TL, Obot Akata C, Shohet R, McDonald JD, Gigliotti A (2007). Gasoline exhaust emissions induce vascular remodeling pathways involved in atherosclerosis. Toxicol Sci.

[b16-ehp-118-921] Lund AK, Lucero JA, Lucas S, Madden MC, McDonald JD, Seagrave JC (2009). Vehicular emissions induce vascular MMP-9 expression and activity via endothelin-1 mediated pathways. Arterioscler Thromb Vasc Biol.

[b17-ehp-118-921] Markhorst DG, Leenhoven T, Uiterwijk JW, Meulenbelt J, van Vught AJ (1996). Occupational exposure during nitric oxide inhalational therapy in a pediatric intensive care setting. Intensive Care Med.

[b18-ehp-118-921] McDonald JD, Barr EB, White RK, Chow JC, Schauer JJ, Zielinska B (2004). Generation and characterization of four dilutions of diesel engine exhaust for a subchronic inhalation study. Environ Sci Technol.

[b19-ehp-118-921] McDonald JD, Barr EB, White RK, Kracko D, Chow JC, Zielinska B (2008). Generation and characterization of gasoline engine exhaust inhalation exposure atmospheres. Inhal Toxicol.

[b20-ehp-118-921] McDonald JD, Doyle-Eisele M, Campen MJ, Seagrave JC, Holmes TD, Lund A (2010). Cardiopulmonary response to inhalation of biogenic secondary organic aerosol. Inhal Toxicol.

[b21-ehp-118-921] McDonald JD, White RK, Barr EB, Zielinska B, Chow JC, Grosjean E (2006). Generation and characterization of hardwood smoke inhalation exposure atmospheres. Aerosol Sci Technol.

[b22-ehp-118-921] Niza S, Jamal HH (2007). Carbon monoxide exposure assessment among toll operators in Klang Valley, Kuala Lumpur, Malaysia. Int J Environ Health Res.

[b23-ehp-118-921] O’Toole TE, Zheng YT, Hellmann J, Conklin DJ, Barski O, Bhatnagar A (2009). Acrolein activates matrix metalloproteinases by increasing reactive oxygen species in macrophages. Toxicol Appl Pharmacol.

[b24-ehp-118-921] Peters A, von Klot S, Heier M, Trentinaglia I, Hörmann A, Wichmann HE (2004). Exposure to traffic and the onset of myocardial infarction. N Engl J Med.

[b25-ehp-118-921] Qureshi MA, Shah NJ, Hemmen CW, Thill MC, Kruse JA (2003). Exposure of intensive care unit nurses to nitric oxide and NO_2_ during therapeutic use of inhaled nitric oxide in adults with acute respiratory distress syndrome. Am J Crit Care.

[b26-ehp-118-921] Ramos C, Cisneros J, Gonzalez-Avila G, Becerril C, Ruiz V, Montaño M (2009). Increase of matrix metalloproteinases in woodsmoke-induced lung emphysema in guinea pigs. Inhal Toxicol.

[b27-ehp-118-921] Restrepo C, Zimmerman R, Thurston G, Clemente J, Gorczynski J, Zhong M (2004). A comparison of ground-level air quality data with New York State Department of Environmental Conservation monitoring stations data in South Bronx, New York. Atmos Environ.

[b28-ehp-118-921] Sarnat JA, Marmur A, Klein M, Kim E, Russell AG, Sarnat SE (2008). Fine particle sources and cardiorespiratory morbidity: an application of chemical mass balance and factor analytical source-apportionment methods. Environ Health Perspect.

[b29-ehp-118-921] Seagrave J, Campen MJ, McDonald JD, Mauderly JL, Rohr AC (2008). Oxidative stress, inflammation, and pulmonary function assessment in rats exposed to laboratory-generated pollutant mixtures. J Toxicol Environ Health A.

[b30-ehp-118-921] Seagrave J, McDonald JD, Bedrick E, Edgerton ES, Gigliotti AP, Jansen JJ (2006). Lung toxicity of ambient particulate matter from southeastern U.S. sites with different contributing sources: relationships between composition and effects. Environ Health Perspect.

[b31-ehp-118-921] Shah AP, Pietropaoli AP, Frasier LM, Speers DM, Chalupa DC, Delehanty JM (2008). Effect of inhaled carbon ultrafine particles on reactive hyperemia in healthy human subjects. Environ Health Perspect.

[b32-ehp-118-921] Sun Q, Wang A, Jin X, Natanzon A, Duquaine D, Brook RD (2005). Long-term air pollution exposure and acceleration of atherosclerosis and vascular inflammation in an animal model. JAMA.

[b33-ehp-118-921] Sun Q, Yue P, Deiuliis JA, Lumeng CN, Kampfrath T, Mikolaj MB (2009). Ambient air pollution exaggerates adipose inflammation and insulin resistance in a mouse model of diet-induced obesity. Circulation.

[b34-ehp-118-921] Sun Q, Yue P, Ying Z, Cardounel AJ, Brook RD, Devlin R (2008). Air pollution exposure potentiates hypertension through reactive oxygen species-mediated activation of Rho/ROCK. Arterioscler Thromb Vasc Biol.

[b35-ehp-118-921] Zhang SH, Reddick RL, Piedrahita JA, Maeda N (1992). Spontaneous hypercholesterolemia and arterial lesions in mice lacking apolipoprotein E. Science.

